# Could the Therapeutic Effect of Physical Activity on Irritable Bowel Syndrome Be Mediated Through Changes to the Gut Microbiome? A Narrative and Hypothesis Generating Review

**DOI:** 10.1111/nmo.70004

**Published:** 2025-03-03

**Authors:** Hannah B. Lindsell, Neil C. Williams, Daniele Magistro, Maura Corsetti, Gemma E. Walton, Kirsty A. Hunter

**Affiliations:** ^1^ Department of Sport, Health and Performance Enhancement (SHAPE) Research Centre, Department of Sport Science Nottingham Trent University Nottingham UK; ^2^ NIHR Nottingham Biomedical Research Centre Nottingham University Hospitals NHS Trust UK, School of Medicine Nottingham UK; ^3^ Department of Food and Nutritional Sciences The University of Reading Reading UK

**Keywords:** gastrointestinal disorder, gut brain axis, gut microbiome, irritable bowel syndrome, physical activity

## Abstract

**Background:**

Irritable bowel syndrome (IBS) is one of the most prevalent gastrointestinal (GI) disorders worldwide. Defined as a disorder of gut‐brain interaction, its pathophysiology is still not completely clear. Consequently, current treatments primarily target symptoms rather than addressing the cause of the condition. The gut microbiome is increasingly acknowledged as central to IBS pathophysiology and, thus, may have therapeutic potential. Several national treatment guidelines recommend increasing physical activity for IBS management.

**Aims:**

This review summarises the evidence about the relationship between physical activity, IBS symptoms, and the gut microbiome, investigating the hypothesis that physical activity's therapeutic effects on IBS may be explained via modulation of the gut microbiome.

**Results:**

This review revealed that routine exercise was associated with a 15%–66% reduction in symptom severity and up to 41% enhanced QoL in IBS participants, and modulates the gut microbiome in healthy controls.

**Discussion:**

This review generates the hypothesis that routine physical activity may favorably alter gut microbiome composition in IBS to improve IBS symptomology. While a plausible hypothesis, research needs to confirm whether gut microbiome modulation is involved in physical activity associated IBS symptom relief.

**Conclusion:**

Furthermore, the establishment of the most effective mode, duration, and intensity of physical activity for each sex and IBS‐subtype is needed, with patient input during this process crucial to successfully translate science into practice.

## Graphical abstract

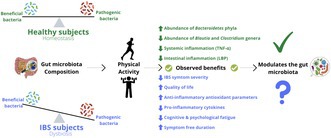

**Abbreviations**
16S rRNA: 16S ribosomal ribonucleic acid
1β: Interleukin 1‐Beta
ASV: amplicon sequence variants
BSG: British Society of Gastroenterology
FGID: functional gastrointestinal disorder
FODMAP: fermentable oligosaccharides, disaccharides, monosaccharides and polyols
GI: gastrointestinal
IBS: irritable bowel syndrome
IBS‐C: IBS with constipation
IBS‐D: IBS with diarrhea
IBS‐M: IBS mixed (both constipation and diarrhea)
IBS‐U: undefined IBS
IL‐6: Interleukin‐6
IL‐8: Interleukin‐8
LBP: lipopolysaccharide binding protein
MICT: moderate intensity continuous training
mRNA: messenger RiboNucleic Acid
NICE: National Institute for Health and Care Excellence
QoL: quality of life
qPCR: quantitative polymerase chain reaction
SCFA: short chain fatty acid
SIT: sprint interval training
TLR‐4: toll‐like receptor 4
TLR‐5: toll‐like receptor 5
TNF‐α: tumor necrosis factor alpha
VO_2peak_
: maximum volume of oxygen uptake during peak exercise
WHO: World Health Organization

**Summary**

- Physical activity is associated with a 15%–66% reduction in IBS symptom severity and up to a 41% improvement in quality of life.
- The gut microbiome plays a potential role in mediating the benefits of exercise for IBS symptom relief.
- Current evidence suggests that regular exercise modulates the gut microbiome in healthy individuals, but more research is needed to confirm these effects in patients with IBS.
- Further studies should determine the optimal mode, duration, and intensity of physical activity for IBS management, considering patient preferences.
- Understanding the gut microbiome's role in IBS treatment could improve non‐pharmacological strategies for symptom relief.

## 1 Introduction

IBS is one of the most commonly diagnosed functional disorders of the gastrointestinal tract, affecting around 3%–5% of the global population [1, 2] depending on the diagnostic criteria used. It is characterised by abdominal pain, with altered bowel habits [3]. Typically diagnosed by the Rome IV Criteria [4], IBS is categorised into 4 sub‐types based upon predominant symptomology; IBS‐D, (diarrhea dominant), IBS‐C (constipation dominant), IBS‐M (mixed, alternating between diarrhea and constipation), and IBS‐U (undefined).

IBS has recently been reclassified from a ‘functional gastrointestinal disorder’ (FGID) to a ‘disorder of gut‐brain interaction’, by the Rome Foundation ([3, 5]), thus emphasising the central role of the gut‐brain axis in the condition. Despite not increasing mortality, IBS has profound personal and socioeconomic impacts including on quality of life (QoL) (Black and Ford 2020) [6], and workplace absenteeism [7] along with treatment costs of ∼£2.07 billion per year in the UK [8].

The pathophysiology of IBS is poorly understood. Although there is an absence of anatomical or biochemical markers to diagnose IBS, several host‐related factors such as alteration in the gut microbiota and gut‐brain interaction, visceral hypersensitivity, altered pain perception, increased intestinal permeability, amplified gut mucosal immune activation and psychological factors are proposed to play a role in contributing to the manifestation of IBS [3, 9]. The microbiome refers to the microorganisms in the gut, their genes and their products [10] as opposed to the term ‘gut microbiota’ which refers purely to the gut microorganisms themselves [11]. Communication along the pathways of the gut‐brain axis facilitates bidirectional modulation of the gastrointestinal tract and central nervous system [12], thus having widespread effects on several physiological processes. For example, ‘top down’ whereby bowel functions are regulated in response to emotions and cognition, and ‘bottom‐up’ whereby gut stimuli influence cognition and emotional centers of the brain. It is plausible that altered gut‐brain communication may be one mechanism by which the gut microbiome affects IBS pathophysiology [13, 14, 15].

There is currently no cure for IBS although a range of strategies are used to manage symptomology and reduce disease burden. These include dietary modification, pharmacological, and non‐pharmacological treatments such as psychological and behavioral therapies. At present, the most effective management strategy has multiple components to address the heterogeneity of the condition and symptomology. In the UK, for example, the National Institute for Health and Care Excellence (NICE) and British Society of Gastroenterologly (BSG) [5] provide dietary, lifestyle and pharmacological management guidelines.

Physical activity is defined as any bodily movement produced by the skeletal muscles that results in energy expenditure, measured in kilojoules (kJ) or kilocalories (kcal). Exercise is a subset of physical activity that is planned, structured and involves repetitive bodily movements, with the purpose of improving physical fitness (a set of attributes that people either have or achieve, which relates to their ability to perform physical tasks) [16]. Studies have observed exercise interventions to modulate the gut microbial composition in healthy controls [17, 18]. Interestingly, many current therapeutic strategies for IBS influence the gut microbiome for example, probiotics, dietary modification, Loperamide, laxatives, non‐absorbable antibiotics, tricyclic acids, and selective serotonin reuptake inhibitors, therefore, it is reasonable to consider whether modulation of the gut microbiome may be a mechanism by which these therapies manage IBS symptoms, particularly given the emerging role of the gut microbiome in the pathophysiology of IBS. Thus, it could be beneficial for IBS therapies to target the gut microbiome. Increasing physical activity via regular exercise may be one of these strategies in IBS.

The focus of this manuscript was to review the literature on the impact of exercise on symptoms and quality of life in IBS and explore whether evidence suggested a possible role of microbiome in modulating this effect. (see Data S1 for literature search strategy).

## 2 Physical Activity and Irritable Bowel Syndrome Management

Seven studies included in this review have investigated the role of exercise in IBS. All have reported various forms of exercise to modulate IBS symptoms and quality of life (QoL) (see Table 1).

**TABLE 1:** Design details and main findings of studies examining the impact of exercise on IBS symptoms and related measures between 2004 and 2024.

Author and Country	Participant characteristics	Intervention type	Frequency	Intensity	Duration	Follow‐up	Mode of delivery	Main findings	RoB
Daley [19] England	56 sedentary subjects (M:15, F:41), 30–56 years IBS subtype not stated	EX: Moderate intensity CON: maintain usual activity	5 days/week	EX: 30 min moderate	12 weeks	12 weeks	Guided	EX vs. CON: 33% reduction in constipation symptoms No significant difference in IBS‐QOL between groups	Mod
Johannesson [20] Sweeden	73 sedentary subjects (F:73) 18–65 years IBS subtype not stated	EX: Increased chosen exercise CON: maintain usual activity	3–5 days/week	Moderate increase	12 weeks	No follow up	Self‐directed	EX vs. CON: ↓ ~16% IBS‐SSS ↑ 17% IBS‐QOL	Mod
Johannesson [21] Sweden	33 already active subjects (Mean: 192 min/wk) (F:33) 28–61 years IBS subtype not stated	Exercise of choice	2 h extra/week	Varies	12 weeks	~5 year	Self‐directed	Baseline vs. Follow‐up: ↓ ~21% IBS‐SSS ↓ Anxiety and depression ↓ Cognitive and psychological fatigue ↑ 21% IBS‐QOL 16% firmer stool	Mod
Hajizadeh Maleki [22] Iran	51 sedentary subjects (F:51) 18–41 years IBS subtype not stated	Walking or Running CON: maintain usual activity	25–30 min/d, 3–4 days/week	45%–55% VO_2max_	24wks	30 and 60 days post	Self‐directed	EX vs. CON: ↓ 29% IBS‐SSS ↑ Anti‐inflammatory and antioxidant parameters ↓ pro‐inflammatory cytokines	Mod
Davydov [23] United States	27 subjects (M:3, F:24) (active < 1 h/d 7d/wk), 23–54 years IBS subtype not stated	Iyengar Yoga and walking programmes	2 days/week	Moderate	16wks	6 months	Guided	Yoga vs. Walking: ↑ symptom free duration in both conditions ↓ Pain severity Walking programme: ↓ GI symptoms	Low
Fani [24] Iran	20 subjects (F:20) (not regularly completing aerobic exercise) 22–43 years IBS subtype not stated	Treadmill exercise CON: maintain usual activity	3 days/week	70%HR_max_	6wks	No follow up	Guided	EX vs. CON: ↓ 66% IBS‐SSS ↑ 41% IBS‐QOL	Low
Riezzo [25] Italy	40 mild–moderate IBS patients (M:11, F:29) (activity level not specified) 18–65 years IBS subtype not stated	Walking	3 days/week	@60%–75% HRmax	12wks	No follow up	Guided	Baseline vs. Follow‐up: ↓ 39% IBS‐SSS ↑ QoL, and Health SF‐36 ↓ symptoms SCL‐90‐R, and Psychophysical QPF/R‐stress.	Low

Abbreviations: CON, control group; EX, exercise; HR, heart rate; IBS‐SSS, Irritable Bowel Syndrome Symptom Severity Scale; QoL, quality of life; RoB, risk of bias.

Of the 7 studies in Table 1 that evaluated the response of IBS symptoms to exercise interventions that increased physical activity using the IBS‐SSS, two studies were from the same group and country [20, 21]. Following a 12‐week prospective exercise intervention in which 33 female IBS‐patients (of unspecified sub‐type) engaged in exercise of their choice, [21] Johanneson et al. reported a 16% reduction in Bristol stool form scale, indicative of a shift towards firmer stool consistencies (Baseline: 4.5, Follow up 3.8, *p* = 0.004), 0 = hard lumps, 7 = watery, which may be interpreted as a positive outcome for IBS‐D patients. This effect may be attributed to exercise stimulating peristalsis in the GI tract, thereby regulating transit time of stools through the intestines, however without specific details of the mode and intensity of exercise it is diffuclt to determine the precise mechanism. Conversely, another 12‐week prospective study by Johannesson et al. [20] in 73 female IBS pateints of undefined subtype observed a non‐significant increase in stool form (Baseline: 4, follow‐up: 5). This suggests that the effects of physical activity on stool form may vary among individuals and the mode of activity completed, and may not always yield a significant improvement. It is pertinent to note that the absence of IBS‐subtype, exercise details, controls or males in either study [20, 21]; is a crucial factor to consider as different IBS‐subtypes can present with varying symptom profiles, and are likely to respond differently to exercise. Thus, tailored exercise prescriptions similar to pharmacological treatments may be needed to optimise effect, and modify exercise prescription to IBS subtype. Therefore, these findings, while promising in terms of stool consistency improvement, should be interpreted cautiously, and additional research is needed to understand how exercise specifically affects different IBS subtypes.

**FIGURE 1:**
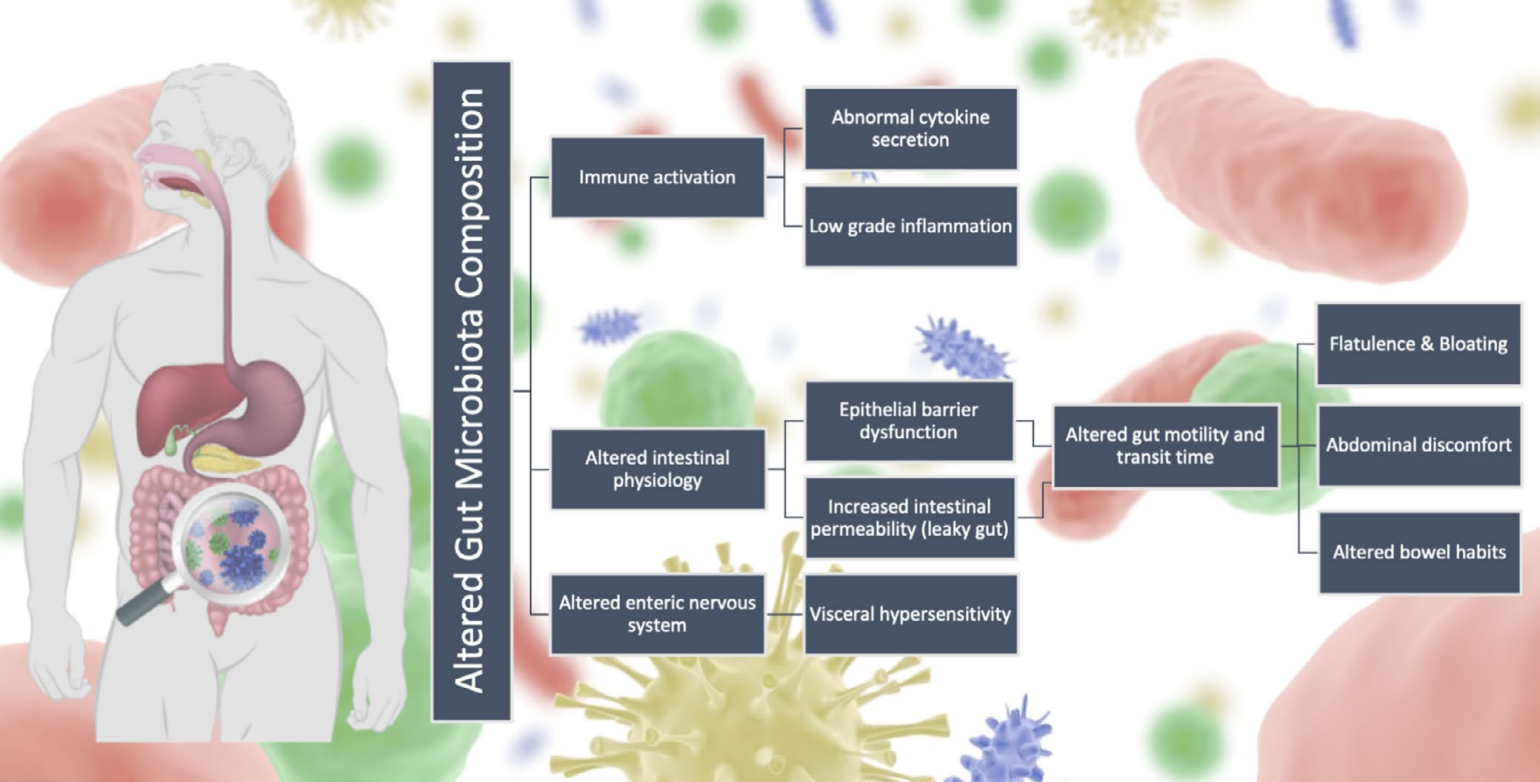
Schematic overview of gut microbiota dysbiosis and associated physiological alterations in IBS.

The five studies that measured QoL reported variable (0%–41%) improvements in QoL scores [19, 20, 21, 24, 25] following exercise interventions. This may be attributed to the variation in type, intensity or duration of exercise completed between studies and whether it was guided or self‐directed, in addition to individual factors such as baseline fitness levels, IBS‐subtype or IBS‐SSS (Table 1). For example, the intensity of the self‐directed exercise interventions [20, 21] may lack the precision of the guided studies whereby activity was guided and intensity was managed by a specific percentage of HRmax [24, 25]. Of the nine IBS‐QoL dimensions, the most recent study observed statistically significant differences between baseline and post intervention in all dimensions except sexual [25], and both Johannesson et al. studies [20, 21] observed statistically significant differences between baseline and post intervention in five of the dimensions; emotional, sleep, energy, physical functioning, and social role. Nonetheless, it is important to further investigate the mechanisms responsible for these positive outcomes and to identify the specific IBS‐subtypes that would benefit the most from exercise interventions.

Some of the previous studies provided insight into the possible mechanism of action whereby physical activity impacts IBS [22]. A 24‐week prospective walking and running intervention in 24 female IBS patients showed an increased in anti‐inflammatory and antioxidant blood parameter activity; superoxide dismutase, glutathione peroxidase and catalase, in addition to reducing pro‐inflammatory cytokines and peroxidative biomarkers (1β, IL‐6, IL‐8 and TNF‐α) when compared to 27 female non‐exercise IBS controls who maintained usual activity [22]. These findings are of interest considering the elevated IL‐6 mRNA levels and higher expression of TLR‐4, TLR‐5, and CXCR‐3 observed among IBS patients compared to controls, particularly in IBS‐D [9].

Davydov et al. [23] observed that the type of exercise influenced baroreceptor sensitivity and baroflex effectiveness differently in a prospective 16‐week study in 27 IBS patients (M:3, F: 24). Nonetheless, both a 16‐week walking programme and Iyenar yoga programme increased symptom free duration in 27 IBS patients (M:3, F:24) [23] (Table 1). This suggests, that while different modes of exercise may alter physiological mechanisms, transit time and the gut‐brain axis, both routine walking and Iyengar yoga can increase symptom free duration, underlining the value of exercise for symptom relief.

Whilst encouraging, the current body of research exhibits notable inconsistencies in study methodologies. This lack of uniformity and consideration of important influential variables such as IBS‐subtype, age, and sex, leaves significant gaps in the existing literature base. Despite this, both the BSG and NICE guidelines suggest that IBS patients should be informed of the importance of physical activity. NICE also suggest that physical activity levels should be assessed, and if low, for example, < 150‐min of moderate‐to‐vigorous‐intensity physical activity per week [26], an increase in physical activity should be encouraged. However, an appropriate frequency, duration, mode, and intensity of exercise is not specified, particularly considering exercise‐induced GI symptoms, which are common in the general population and athletes [27], may be exacerbated or experienced differently in IBS patients.

Moreover, both NICE and BSG do not differentiate between IBS‐subtypes with their physical activity recommendations despite doing so for pharmacological therapies for example, Loperamide for IBS‐D and linaclotide for IBS‐C.

Consequently, there is currently insufficient evidence for practitioners to prescribe physical activity as an adjunct IBS management strategy, particularly as the ideal would be to tailor exercise prescription which takes into consideration individual characteristics (IBS‐subtype, sex, and age) to match the patient's specific needs.

## 3 IBS And the Gut Microbiome

The human GI tract houses a diverse microbial community, comprising trillions of microorganisms (bacteria, archaea, viruses, and fungi) [28]. Notably Firmicutes and Bacteroidetes collectively constitute 90% of the gut bacteria [29]. The gut microbiota plays a pivotal role in digestion, metabolism [30], immune function [31], and gut‐brain communication through the nervous, immune and endocrine systems [32]. A diverse gut microbiota is recognised to positively correlate with optimal health [33], while disruption to the gut microbiota, often referred to as ‘dysbiosis’ [34], has been implicated in the pathophysiology or numerous conditions, including IBS [35, 36]. Consequently, there is growing interest in the prophylactic and therapeutic potential of modulating the human gut microbiota through pre‐, pro‐, syn‐ and post‐biotic supplementations, and fecal microbiota transplants [37].

IBS is associated with alterations in the gut microbiome composition and function; however, precise microbial patterns are yet to be identified, particularly across different IBS subtypes and sexes. Bacteria such as *Bacteroides*, *Faecalibacterium prausnitzii* , *Ruminococcus spp*., and *Bifidobacteria* have been implicated in IBS, however longitudinal omics studies have not uncovered uniform characteristics in the IBS gut microbiota, instead these studies reveal significant variability between individuals and over time [38]. There are methodological disparities in current research such as study design and uncontrolled cofounders like diet, which hinder accurate characterisation of the gut microbiota in IBS. Furthermore there are limitations in 16S rRNA sequencing in cross‐sectional studies, as a single fecal sample may not accurately reflect gut microbiota composition. Longitudinal studies are needed to address this and assess changes over time [39].

The mechanisms responsible for modulation of the gut microbiota in IBS remain unclear. Reports in germ‐free mice suggest that the gut microbiota plays a role in the pathophysiology via altered gut‐brain communication [40]. Nonetheless, whether gut microbiota changes are a cause or effect of IBS remains unclear, however there are links between IBS‐associated dysbiosis and physiological alternations such as immune function with abnormal cytokine secretion (TNF‐a and IL‐6) [41], low‐grade inflammation [42], epithelial barrier dysfunction [43] and increased intestinal permeability [44] (Figure 1). These changes may impact gut motility, transit time and symptoms [43] such as flatulence, abdominal discomfort, altered bowel habits, and visceral hypersensitivity [45]. The gut microbiota is inceasingly recognised to contirubte to visceral hypersensitivity [46, 47], which is characterised by hyperalgesia and allodynia [48], possibly through its influence on enteric nervous system dysfunction and increased intestinal permeability [49].

## 4 Physical Activity and the Gut Microbiome

The multifaceted relationship between the gut microbiota, physical activity and disease pathophysiology is gaining attention, though this is an emerging area of research with limited studies abailable. Highly active individuals, including athletes, tend to exhibit increased gut microbial diversity, attributed in part to physical activity's influence on microbial composition [50, 51, 52] (Table 2). Furthermore, studies in which exercise was prescribed in doses recommended by the World Health Organisation (WHO), have demonstrated diversification of the gut microbiota and associated health improvements in both healthy and diseased populations [53, 54] (Table 2).

**TABLE 2:** Design details and key findings of studies investigating the relationship between exercise and sedentariness and the gut microbiota in adults between 2004 and 2024.

Author and country	Participant characteristics	Type of intervention	Duration	Frequency	Intensity	Mode of delivery	Microbiome Measurements	Main findings	RoB
Motiani [18] Finland	26 sedentary subjects, (M:16, F:10), Insulin resistant 40–55 years	SIT vs. MICT	2 weeks	3 weeks	SIT: 10% FFM, MICT: 60% VO_2peak_	Randomly assigned and Guided	Stool samples Real‐Time qPCR analysis of 16S rRNA gene sequencing	SIT and MICT Baseline vs. Follow‐up: ↓ TNFα, ↓ Body fat %, ↑ Abundance of Bacteroidetes phyla ↓ Firmicutes/Bacteroidetes ratio, ↓ Relative abundance of *Blautia* and *Clostridium* genus. Post SIT INV: ↑ VO_2peak_ Post MICT INV: ↓ LBP	Low
Jollet [55] France	18 healthy subjects (M:18) (activity level not specified) 26–40 years	DI with counter‐pressure thigh cuffs vs. CON	4 days baseline, 5 days DI, 2 days recovery	N/A	Thermo‐neutral	Guided	Stool sample Real‐Time qPCR analysis of 16S rRNA gene sequencing	DI vs. CON: ↓ Whole body and lean leg masses, Bacteroidetes, Firmicutes, Proteobacteria abundance unchanged, ↑ Clostridiales order abundance and Lachnospiraceae family (Firmicutes phylum) ↓ Fecal propionate conc	Low
Resende [56] Brazil	24 sedentary subjects (M:24) 20–45 years	EX vs. CON	10 weeks	3 weeks	60%–65% VO_2peak_	Guided	Stool sample Real‐Time qPCR analysis of 16S rRNA gene sequencing	EX vs. CON: ↑ Cardiorespiratory fitness (VO_2peak_) ↑ *Streptococcus* genus, ↓Clostridiales‐order	Low
Moitinho‐Silva [57] Germany	42 inactive subjects 20–45 years 13 elite athletes (Fecal sample only)	END (30 min run) vs. STR (30 min whole body) vs. CON (maintain usual activity)	9 weeks	3 weeks	> 85% HR_max_	Randomly assigned and Guided	Stool sample Real‐Time qPCR analysis of 16S rRNA gene sequencing	END: ↓ estimated number of different bacterial taxa vs. STR and CON. Intervention had no significant effects on community diversity, structure or abundance in any group. Difference in baseline ASV abundance between inactive and athletes	Low
Bycura [17] San Francisco	56 healthy subjects, (M: 18, F:38) 18–33 years (activity level not specified)	CRE vs. RTE	8 weeks	3 weeks	CRE: 60%–90% HR_max_ RTE: 70%–85% 1RM	Guided	Stool sample Real‐Time qPCR analysis of 16S rRNA gene sequencing	CRE: Altered gut microbiota composition however changes did not persist after completion of exercise. RTE: minimal effect on gut microbiota composition.	Low

*Note:* Excludes studies that combined the effects of dietary, and exercise induced modulation of the gut microbiota.

Abbreviations: 1RM, one‐repetition maximum; ASVs, amplicon sequence variants; BM, body mass; BP, blood pressure; CON, control Group; conc, concentration CRE (cardiorespiratory exercise); DI, dry immersion; END, endurance; FFA, free fatty acid; FFM, fat free mass; HR, heart rate; INV, intervention; MICT, moderate‐intensity continuous training (sustained exercise without rest); RMR, resting metabolic rate; RoB, risk of bias; rRNA, ribosomal ribonucleic acid; RTE (resistance training exercise); SIT, sprint interval training; STR, strength.

The findings in Table 2 suggest that increased routine exercise could modulate taxonomical abundances of bacteria in the gut microbiota. A prospective study by Motiani et al. [18] among 26 healthy adults (M:16; F: 10), reported an increase in the relative abundance of Bacteroidetes phyla following 2‐weeks of Sprint Interval Training (SIT) or Moderate Intensity Continuous Training (MICT) alongside a reduction in the Firmicutes/Bacteroidetes ratio, but no change in bacterial richness or diversity. These observations are noteworthy as it is widely accepted that Bacteroidetes are reported to be lower in relative abundance among IBS patients, thus perhaps routine exercise could be an effective strategy to restore the relative abundance of Bacteroidetes in their gut microbiota.

Motiani et al. [18] also found that 2‐weeks of SIT or MICT exercise reduced the relative abundance of the genera *Blautia* and *Clostridium*, which is consistent with previous research [58, 59]. *Blautia* prevalence is positively correlated with TNF‐α, a pro‐inflammatory cytokine, while *Clostridium* influences immune function and maintenance of intestinal homeostasis [60, 61]. Specifically, Clostridial strains are reported to induce cytokine production (TNF‐α, IL‐10, and IL‐8), and the relative abundance of clostridia is known to drive inflammation [62]. Therefore, reduction in the relative abundance of *Clostridium* may be one mechanism whereby the exercise intervention was associated with reduced plasma concentrations of TNF‐α, and Lipopolysaccharide Binding Protein (LBP) [18, 56] (Table 2).

Resende et al. [56] observed a positive correlation between increased routine exercise (from sedentary to 150‐min per week at 60%–65% VO_2peak_) and mean relative abundance of *Streptococcus* genus, and a decrease in Clostridiales order in 24 previously sedentary men, following a 10‐week aerobic exercise training intervention whilst their habitual diet was unchanged. This prospective study further supports the notion that routine exercise can have modulatory effects upon the gut microbiota.

Physical inactivity (insufficient moderate‐vigorous physical activity) and sedentary behavior, ≤ 1.5 Metabolic equivalent of tasks [26] are interesting constructs which are the opposite of physical activity. Hence it is important to identify whether they impact the gut microbiome and, ultimately, if they are also risk factors for IBS. In a severe hypoactivity model (five‐days of sedentary DI) in 18 healthy men, a metagenomic quantitative polymerase chain reaction (qPCR) analysis showed an increase in the relative abundance of Clostridiales order by 3.8%, specifically the Lachnospiraceae family by 3.9%, which could have negative consequences for human health and lead to dysbiosis, contributing to GI disorders such as IBS [55]. While Lachnospiraceae is generally considered to be a beneficial anaerobe from the Firmicutes phylum, different species within this family have been associated with intra‐ and extraintestinal diseases (Crohn's disease, IBD, major depressive disorder, metabolic syndrome, obesity, type 2 diabetes) [63, 64, 65, 66], thus, it is possible that sedentary behavior may unfavorably increase the abundance of taxa detrimental to health. Clostridiales order encompasses both commensal and opportunistic species, however the specific family, genus or species profiles remains ambiguous in many studies [18, 55, 56] complicating the interpretation of its impact on human health, further necessitating the need for multi‐omics studies. It is possible that inactive participants [55] may have developed an increase in opportunistic Clostridiales abundance, potentially contributing to dysbiosis and disease associated with inactivity. Conversely, routine activity likely resulted in the reduction of opportunistic Clostridiales abundance [56], possibly yielding health benefits. However, further research is warranted, including the application of shotgun metagenomics rather than 16S rRNA sequencing or qPCR analysis. This approach would allow for identification of species‐level changes within the Clostridiales order and to enable more information to be gathered on their respective roles in gut microbiota dynamics and human health. This 5‐day DI sedentary intervention also induced a reduction in fecal concentrations of the beneficial SCFA, propionate, but had no effect on butyrate or acetate [55] (Table 2). Whilst this is an extreme model and may not be a direct match for sedentary IBS patients, this does provide valuable insight. IBS patients can experience a high level of functional impairment which can contribute to a more sedentary lifestyle, as individuals with IBS frequently reduce their activity levels to manage symptoms [4, 67].

Moreover, Moitinho‐Silva et al. [57] found differences in the abundance of Amplicon Sequence Variants (ASVs) between 42 physically inactive individuals and 13 elite athletes but no difference in total ASV occurrences, in a prospective 9‐week study. For example, physically inactive subjects exhibited a greater abundance of *Dialister, Odoribacter* and *Phascolarctobacterium* genera compared to elite athletes [57]. Notably, *Phascolarctobacterium* has been positively correlated with enhanced mood [68]. Conversely, elite athletes presented with a significantly greater abundance of *Parasutterella*, the family Ruminococcaceae and the beneficial bacteria, *Coprococcus* compared to inactive participants. However, research to elucidate the precise roles of these bacteria in health and disease is sparse. Thus, the findings of both Silva et al. (2021) and Jollet et al. [55] support the notion that not only physical activity, but also physical inactivity, can influence the composition of the human gut microbiota [55, 57]. This could be useful information for those suffering from IBS, assuming the effects of exercise on the gut microbiota are similar in IBS patients and healthy individuals.

### 4.1 Mechanisms for Physical Activity Induced Modulation of the Gut Microbiota

The mechanisms responsible for the observed changes to the gut microbiota community with physical activity are not understood. Nonetheless, acute exercise induces ischemia, heat stress, metabolic flux, gut barrier resilience, and increased gut motility which could plausibly be involved [69, 70, 71].

Moving beyond acute exercise there is a noticeable lack of research exploring the mechanisms underlying how routine exercise affects the gut microbiota, as seen in Table 2. Potential mechanisms include reduced inflammation, altered gut motility/transit time, stress reduction, modulation of gut hormones and dietary changes [72, 73]. However, it is important to note that the intensity of physical activity may alter the adaptation seen. For example, studies have observed 6‐weeks of high‐intensity exercise to increase stress and inflammation [72] which may be detrimental to IBS patients, thus moderate exercise may have more suitable effects.

Nonetheless, to gain an accurate mechanistic understanding of how routine exercise modulates the gut microbiota composition, research needs to move to long‐term intervention/cause‐and‐effect studies, inclusive of multi‐omics analysis.

## 5 Discussion

### 5.1 Overview

The aim of the present review was to critically evaluate the existing knowledge about the relationship between IBS, exercise and the gut microbiome. By identifying gaps in current knowledge, this review provides potential directions for future research, with the aim to advance our comprehension and management of IBS.

### 5.2 Limitations of Current Treatments and Recommendations

An incomplete understanding of the pathophysiology of IBS means that IBS treatments have only relatively modest effectiveness, likely attributable to; insufficient academic funding, negligible mortality, stringent criteria for therapy approval, and stigma surrounding IBS. These factors may lead to delays in seeking medical care, misdiagnosis and a slower pace of discovery, leaving limited options for patients (Lea and Whorwell; [5, 74]).

Overall, the advice about physical activity to manage IBS is vague, with insufficient information for patients, and health providers, to follow. Furthermore, the mechanistic understanding of how exercise improves IBS symptomology is poorly understood and the quality of evidence is weak, thus requiring further investigation.

### 5.3 Summary of Findings

**FIGURE 2:**
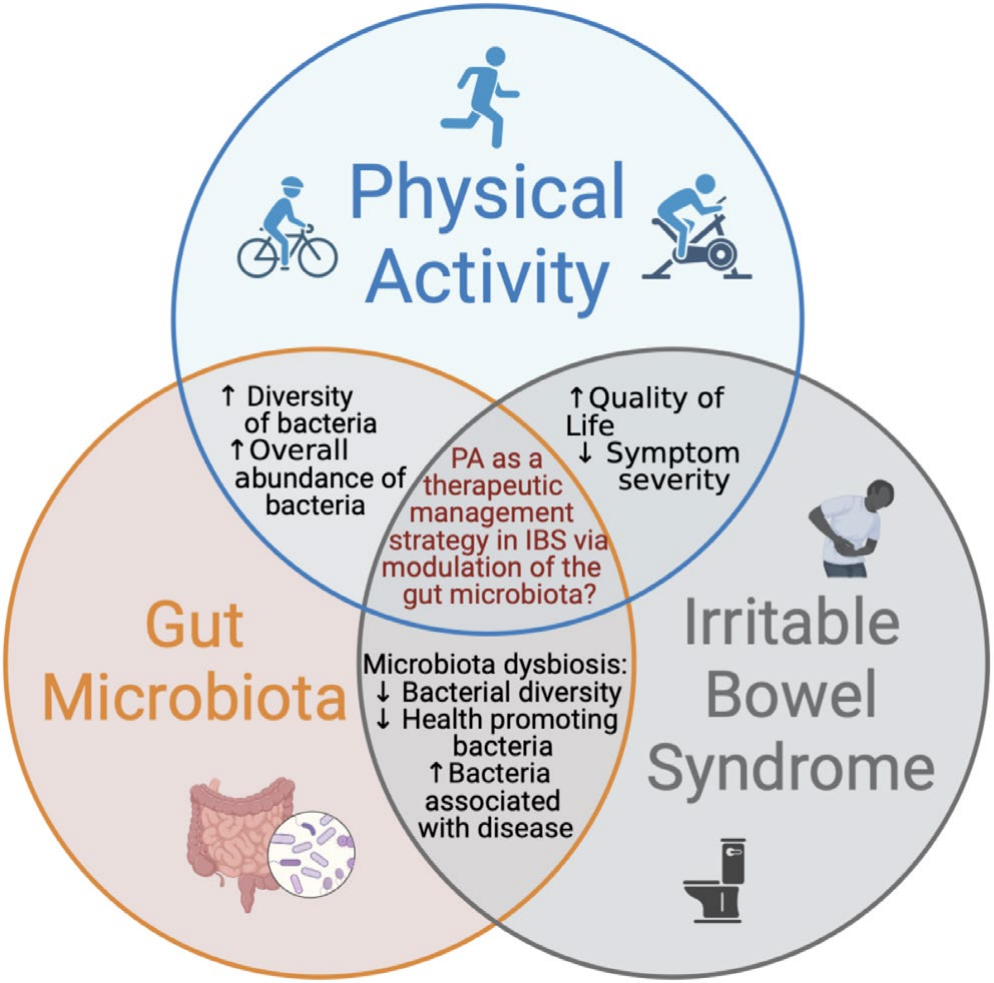
Illustration of the proposed multifaceted relationship between physical activity, the gut microbiota and irritable bowel syndrome. Created with BioRender.com. ↓ decrease, ↑ increase.

The current review acknowledges that whilst there is lack of literature, the studies do demonstrate that exercise can improve IBS symptoms and QoL. Although there are limitations and potential confounding factors such as small sample sizes, dietary variations, and no differentiation between IBS subtypes among others these variations must be considered, as they likely influence IBS outcomes and treatment efficacy. The literature also demonstrates that exercise affects the composition of the gut microbiota in healthy populations but similarly, there are few studies and these are limited by their reliance on a single fecal sample and uncontrolled cofounders like diet. Whether exercise also affects the gut microbiota in IBS is currently unknown and warrants further investigation (Figure 2).

**FIGURE 3:**
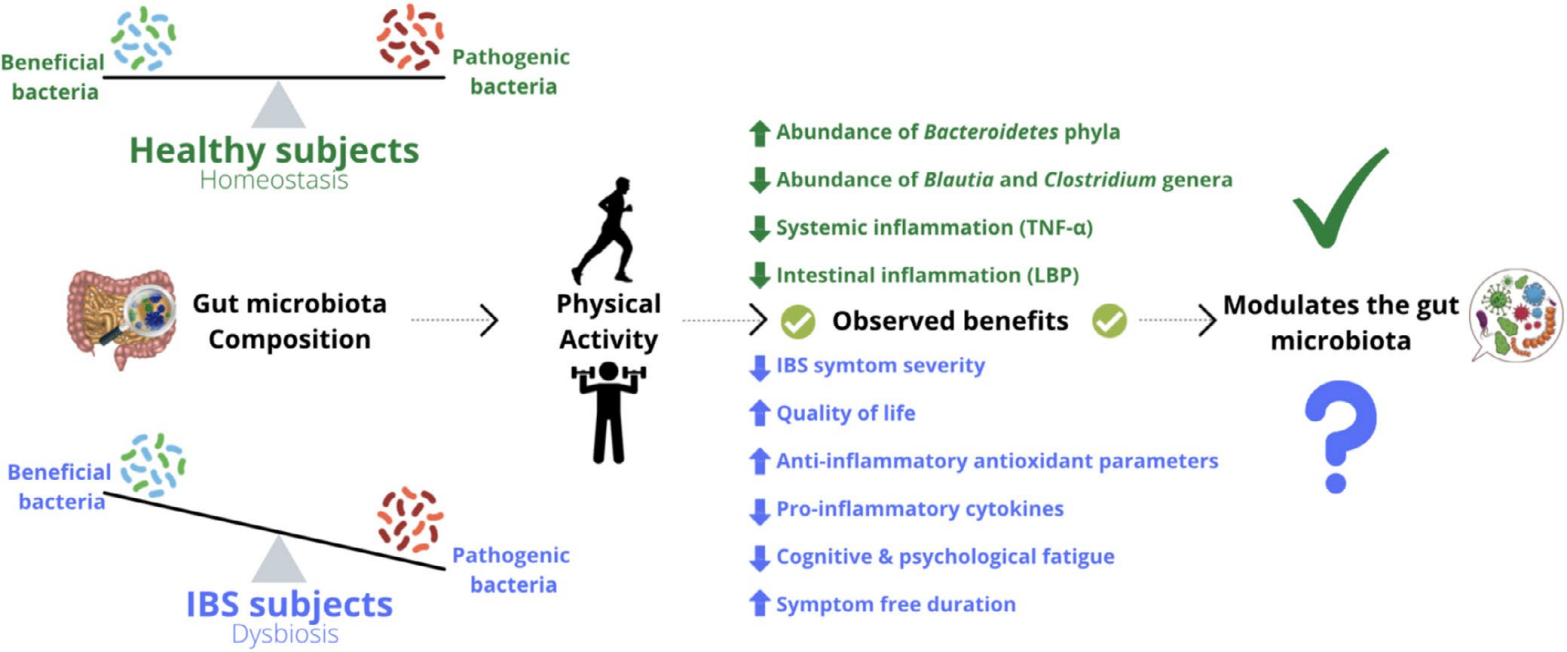
Schematic illustrating the effects of physical activity in healthy controls and IBS patients. LBP, lipopolysaccharide binding protein; TNF‐α, tumor necrosis factor alpha.

Physical activity is advocated as an adjunct management strategy for a range of diseases due to its physiological and psychological benefits, including reduced depression [75]. This may be relevant for IBS, a disorder characterised by altered gut‐brain communication alongside psychiatric (anxiety and depression) and physiological symptomology. Given that multi‐modal IBS management strategies such as a low FODMAP diet, alongside probiotics may work by influencing the gut microbiota, it is possible that also increasing routine exercise may also impart modulatory effects upon the gut microbiota of IBS patients, as has been shown for healthy controls (Table 2 and Figure 3).

### 5.4 Need for Clinical Evidence

Exercise induced modulation of the gut microbiota may be a mechanism through which exercise relieves IBS symptomology. Nonetheless, clinical evidence is required to support this proposition and there are no published studies to date to substantiate this theory. In addition to this, the optimal mode, duration, intensity, and frequency of physical activity to minimise exercise‐induced GI complaints also needs elucidating. Moreover, IBS subtypes vary, and tailored physical activity programs similarly to existing tailored pharmacological therapies (Loperamide, laxatives) may be necessary to address individual symptomology effectively. Given the diverse symptom profiles in IBS, a personalised approach to management is likely to be more effective for clinical efficacy rather than a one‐size‐fits‐all approach.

### 5.5 Future Research Directions

To establish and prescribe effective personalised exercise for managing IBS symptomology, it is crucial to gather insights from individuals with IBS about what exercise would be acceptable. There is only one study which provides this insight [76] and greater patient involvement is needed to allow for successful translation of science into practice. With this intelligence clinicians and reserachers will be able to better understand how interindividual differences affect physical activity's role in IBS management and be better positioned to prescribe appropriate physical activity for IBS patients. It will also support the justification of reserach design to evaluate how the composition and function of the gut microbiota responds to exercise interventions in IBS patients. Future mechansitc exploration should take a multi‐omics approach incorporating, for example, genomics, proteomics and/or metabolomics.

## 6 Conclusions

The relationship between exercise and the gut microbiota is an emerging topic that requires further exploration. While there is speculation that exercise may be an appropriate therapeutic strategy for healthcare practitioners to recommend, reducing IBS symptom severity by up to 66%, the mechanistic evidence and understanding for symptom relief is sparse. However recognising exercise as an adjunct option to pharmacological and other non‐pharmacological strategies is crucial for its effective deployment in clinical practice. This concept represents a novel and significant advancement in the field of IBS management. Studies in healthy controls have suggested that routine exercise can modulate the gut microbiota, however, this has not been extensively studied in IBS‐patients. In addition, the specific mechanisms underlying exercise‐induced gut microbiota modulation are not well understood across populations. Notably, existing literature on the use of exercise to manage IBS symptoms often overlooks the impact of exercise on the gut microbiota and how it interacts with the altered microbial profiles observed in IBS patients. This hypothesis therefore supports the need for research to investigate the efficacy and mechanisms of action of various types of exercise for specific IBS subtypes and whether these vary according to sex. IBS patient participation in the design of these studies is paramount to allow the development of exercise strategies which are acceptable to those with as well as effective.

## Author Contributions

H.B.L. wrote the manuscript. N.C.W., K.A.H., and M.C. provided significant contributions to the conceptualisation, review and editing of the manuscript. D.M. and G.E.W. contributed to the review design and critically reviewed the manuscript. All authors read and approved the final version of the manuscript.

## Conflicts of Interest

The authors declare no conflicts of interest.

## Supporting information


Data S1.

Data S2.

## Data Availability

Data sharing is not applicable to this article as no new data were created or analyzed in this study.
