# Supplementary material for: Could the Therapeutic Effect of Physical Activity on Irritable Bowel Syndrome Be Mediated Through Changes to the Gut Microbiome? A Narrative and Hypothesis Generating Review
Source: Neurogastroenterol Motil. 2025 Mar 3;37(6):e70004. doi: 10.1111/nmo.70004 (PMC12075915; doi:10.1111/nmo.70004)
Supplement: Supplementary file 2 — Data S2. [file NMO-37-e70004-s001.pdf]

Supplementary Materials

1. **Methods:**

This narrative review synthesis recent findings on the relationship between physical activity (PA) the gut microbiome and Irritable Bowel Syndrome (IBS).

2.1 Literature search strategy

A comprehensive literature search was conducted across multiple electronic databases, including PubMed, Library OneSearch, Google Scholar, Web of Science and Science Direct, from December 2021 to August 2024. The search focused on studies published in English within the last 20 years, using the following terms: "irritable bowel syndrome" OR "IBS" AND "physical activity" OR "exercise" OR "sport" OR "fitness."

In addition, randomized controlled trials (RCTs) examining the interaction between physical activity and the gut microbiota were specifically searched in PubMed. To minimise the risk of omitting relevant studies, the reference sections of original articles and reviews were manually screened. All articles that had a title or abstract which looked to meet eligibility criteria were retained for a thorough full text examination.

2.2 Study selection

2.2.1 Eligibility Criteria

The population, intervention, comparison, outcome, and study (PICOS) model was used to establish the eligibility criteria for studies identified through the literature search, as outlined in Table 1.

Table 1. PICOS table detailing criteria for the inclusion and exclusion of studies for the systematic search on PA and IBS.

Property Name	Keywords
Population	Adults (18-65 years) with clinically diagnosed IBS
Intervention	Any method of physical activity
Comparison	Control group/no PA vs physically active. Different PA interventions
Outcomes	No restrictions were placed on outcome measures. Findings were recorded and reviewed reliably
Study Type	Randomised Controlled Trials
Exclusion Criteria	Papers published before 2004<br>Animal or In vitro studies<br>Interventions lasting <6 weeks<br>Studies written in languages other than English<br>Reviews or meta-analyses.<br>Studies including conditions that could alter the composition of the gut microbiota

From the literature search, seven RCTs exploring the relationship between physical activity and IBS were identified in adults (≥ 18 years). Additionally, five studies that examined the

interaction between PA/inactivity and the gut microbiota in adults (≥ 18 years) were identified, all through PubMed, Library OneSearch, Google Scholar, Web of Science and Science Direct databases.

2.2.2 Data Extraction and Analysis

Data from the selected studies was extracted using a standardised form, to ensure consistency and reliability. This captured essential information including study identification, characteristics, intervention details, outcome measures, and key findings. By employing this structured approach, we aimed to facilitate a reliable synthesis of results related to the interaction between physical activity, the gut microbiota, and IBS.

2.2.3 Quality Assessment

The quality of the included studies was assessed using the Cochrane Risk of Bias tool for RCTs. Each study was evaluated based on criteria including randomisation, blinding, and handling of dropouts. This assessment helped determine the reliability of the findings and their implications for clinical practice.

Table 2. Risk of Bias Assessment for Studies examining the impact of PA on IBS symptoms and related measures between 2004 – 2024.

Domain	Daley (2008)	Johannesson (2011)	Johannesson (2015)	Hajizadeh Maleki (2018)	Davydov (2019)	Fani (2019)	Riezzo (2023)
**Random Sequence Allocation**	Low	Low	Low	Low	Low	Low	Low
**Allocation Concealment**	Unclear	Unclear	Unclear	Low	Low	Low	Low
**Blinding of Participants and Personnel**	High	Unclear	High	High	Unclear	Unclear	Unclear
**Blinding of Outcome Assessors**	Unclear	Unclear	High	Unclear	Unclear	Unclear	Unclear
**Incomplete Outcome Data**	Low	Low	Unclear	Low	Low	Low	Low
**Selective Reporting**	Low	Unclear	Low	Low	Unclear	Low	Low
**Other Sources of Bias**	Low	Low	Low	Low	Low	Low	Low

Table 3. Risk of Bias Assessment for Studies examining the relationship between physical activity/inactivity and the gut microbiota in adults between 2004 – 2024.

Domain	Motiani (2020)	Jollet (2021)	Resende (2021)	Moitinho-Silva (2021)	Bycura (2021)

**Random Sequence Generation**	Low	Low	Low	Low	Low
**Allocation Concealment**	Low	Low	Low	Low	Low
**Blinding of Participants and Personnel**	Unclear	Unclear	Unclear	Unclear	Unclear
**Blinding of Outcome Assessors**	Unclear	Unclear	Unclear	Unclear	Unclear
**Incomplete Outcome Data**	Low	Low	Low	Low	Low
**Selective Reporting**	Low	Low	Low	Low	Low
**Other Sources of Bias**	Low	Low	Low	Low	Low

2.2.4 Limitations

This review acknowledges potential limitations, including the variability in study designs, outcome measures, and definitions of physical activity across the included studies. Additionally, the exclusion of non-English studies may limit the comprehensiveness of the findings.
